# Contributing factors of birth asphyxia in Thailand: a case–control study

**DOI:** 10.1186/s12884-023-05885-y

**Published:** 2023-08-15

**Authors:** Panida Rattanaprom, Ameporn Ratinthorn, Siriorn Sindhu, Chukiat Viwatwongkasem

**Affiliations:** 1 Doctoral Candidate, Faculty of Nursing, Mahidol University, Bangkok, Thailand; 2 Faculty of Nursing, Mahidol University, Bangkok, 10700 Thailand; 3 Department of Biostatistics, Faculty of Public Health, Mahidol University, Bangkok, 10400 Thailand

**Keywords:** Birth asphyxia, Intrapartum care service, Quality of care

## Abstract

**Background:**

Birth asphyxia is of significant concern because it impacts newborn health from low to severe levels. In Thailand, birth asphyxia remains a leading cause of delayed developmental health in children under 5 years old. The study aimed to determine the maternal, fetal and health service factors contributing to birth asphyxia.

**Methods:**

A case–control design was conducted on a sample of 4256 intrapartum chart records. The samples were selected based on their Apgar scores in the first minute of life. A low Apgar score (≤ 7) was chosen for the case group (852) and a high Apgar score (> 7) for the control group (3408). In addition, a systematic random technique was performed to select 23 hospitals, including university, advanced and secondary, in eight health administration areas in Thailand for evaluating the intrapartum care service. Data analysis was conducted using SPSS statistical software.

**Results:**

The odds of birth asphyxia increases in the university and advanced hospitals but the university hospitals had the highest quality of care. The advanced and secondary hospitals had average nurse work-hours per week of more than 40 h. Multivariable logistic regression analysis found that intrapartum care services and maternal–fetal factors contributed to birth asphyxia. The odd of birth asphyxia increases significantly in late–preterm, late–term pregnancies, low-birth weight, and macrosomia. Furthermore, maternal comorbidity, non-reassuring, and obstetric emergency conditions significantly increase the odd of birth asphyxia. In addition, an excellent quality of intrapartum care, a combined nursing model, low nurse work-hours, and obstetrician-conducted delivery significantly reduced birth asphyxia.

**Conclusion:**

Birth asphyxia problems may be resolved in the health service management offered by reducing the nurse work-hours. Excellent quality of care required the primary nursing care model combined with a team nursing care model. However, careful evaluation and monitoring are needed in cases of comorbidity, late–preterm, late–term pregnancies, low-birth weight, and macrosomia. Furthermore, increasing the obstetrician availability in obstetric emergencies and non-reassuring fetal status is important.

**Supplementary Information:**

The online version contains supplementary material available at 10.1186/s12884-023-05885-y.

## Introduction

Birth asphyxia is the failure to initiate and sustain spontaneous breathing at birth, causing permanent brain cell damage and threatening the newborn's life. The recovery process requires lengthy hospital observation and intensive care, and may take a lifetime [1–4]. Birth asphyxia is still the leading cause of neonatal morbidity and mortality and remains a significant cause of delayed developmental health in children under 5 years old worldwide. Previous studies reported that maternal–fetal factors affected birth asphyxia [5, 6]. In addition, the appropriate health care service components, such as human resource allocation, specialist availability, competency improvement and an experienced provider contributing to care quality, also impact neonatal outcomes [7]. Therefore, identifying the intrapartum health service factors and maternal–fetal factors contributing to birth asphyxia in Thailand will help to improve the quality of intrapartum care and save neonatal lives.

Birth asphyxia is a significant concern in Thailand due to its higher birth rate (16.0 per 1000 live births in 2021) compared to developed countries (1.5 per 1000 live births) [8, 9]. The range of birth asphyxia rates varies across hospitals in Thailand according to the different levels of hospital. According to health statistics in 2017, the average number of birth asphyxia cases was 40.64 per 1000 live births in advanced hospitals, 41.68 per 1000 live births in university hospitals and 13.42 per 1000 live births in secondary hospitals [10]. These figures show that different hospital types might have different associated factors that affect birth outcomes.

Previous studies reported that maternal–fetal factors affect birth asphyxia such as maternal age, BMI, gestational age, ANC visit, maternal health and obstetric complications [8, 11–14]. Furthermore, the literature reviews reported that healthcare service factors are associated with maternal and neonatal health outcomes such as unequally healthcare providers allocation, nurse staffing, nurse workload, nurse work-hours, nurse-to-patient ratio, and provider performance improvement [15–22]. The appropriate health service resources provide quality intrapartum care. The quality of care during the intrapartum period is crucial to reducing neonatal morbidity and mortality. The two main processes of intrapartum care are the initial evaluation and risk screening and also intrapartum monitoring and care. The initial evaluation and risk screening includes the main strategies recommended for intrapartum practice guidelines during admission. Intrapartum monitoring and care includes labour progress monitoring by using the partograph and close monitoring of the fetal heart rate. All intrapartum women should receive immediate care to reduce the chance of complications during delivery, such as encouraging an upright position, lying on the left side and relaxation. Furthermore, the appropriate duration of expulsion management and an upright position during the second stage provided a positive outcome. The team activated immediately after detecting a severe non-reassuring fetus would resolve the problem of severe hypoxic-ischaemic encephalopathy or perinatal death [23–25]. Timely and appropriate intervention by the in-utero resuscitation technique during non-reassuring fetal status includes changing maternal position, oxygen administration, uterine relaxation and an intravenous fluid bolus to reduce severe hypoxia [23, 26–28].

Moreover, regarding the model of intrapartum care, literature reviews indicated that continuing care or the primary nursing care model was beneficial to perinatal outcomes and reduced neonatal death rates [29–33]. Although the primary nursing care model has better outcomes than the team nursing care model, most labour and delivery care units in Thailand employ the team nursing model or a combined primary nursing care and team model due to provider shortage. However, some reports showed that the team nursing model left tasks undone and decreased patient safety and care quality. In addition to the care model, a professional healthcare team of labour and delivery is also important.

Despite a growing research interest in human resources and health outcomes, there is still a lack of evidence on the effects of healthcare service factors on birth asphyxia. Most research has focused on maternal and fetal factors, with less attention given to hospital factors and birth asphyxia. Therefore, this case–control study was aimed at determining intrapartum health services and maternal–fetal factors that contribute to birth asphyxia at three levels of hospital: university, advanced and secondary. In addition, it is hoped that the research findings will serve as an evidence base for developing national strategic proposals for improving maternal and fetal health outcomes and solving the disparity among intrapartum health services in Thailand.

## Methods

The case–control study was designed to collect data from 4256 intrapartum care charts recorded in 23 hospitals in Thailand from 2016 to 2017. Pregnant women of gestational age 34^+0^–41^+6^ weeks, admitted with signs of labour onset and delivered with a low Apgar score (≤ 7) at the first minute of life, were selected as the case group (852) and those with an Apgar score of > 7 as the control group (3408). Month and type of delivery were matched in the case and control groups. Pregnant women with twin or congenitally abnormal fetuses were excluded.

The sample size was determined from the study by Berazategui et al. [34]. The number of antenatal care visits was selected as a variable and calculated in the SMART program. For a two-sided test with a 5% type I error, the study required a sample size of at least 852 to make this comparison with 80% power. Therefore, the research proportion of case and control samples was 1 to 4 [35]. The overall research sample size was 4260 when the number of control samples was 3408.

The number of estimated hospitals required for collecting data was based on a multilevel research design. The standardized proportion difference as an effect measure was applied to calculate the number of research settings needed to conduct the research [36].

$$d = \frac{\left[{p}_{0}- {p}_{1}\right]}{\sigma pooled}$$

$${\sigma }^{2}pooled = \frac{{n}_{0}{p}_{0}\left(1-{p}_{0}\right) +{n}_{1}{p}_{1}\left(1- {p}_{1}\right)}{{n}_{0}+ {n}_{1}}$$

$$variance\;of\;d\;\leq\left(\frac d{z_{\alpha/2}+z_\beta}\right)^2$$

$$n_j=\frac{4\left[\rho+(1-\rho)/n_i\right]}{varience\;of\;d}$$

where.

*d* = proportion difference as an effect size

*p*_0_ = proportion of birth asphyxia in secondary hospitals (0.011)

*p*_1_ = proportion of birth asphyxia in primary hospitals (0.0068)

σ_pooled_ = pooled standard error of birth asphyxia

*n*_0_ = number of cases of birth asphyxia in secondary hospitals (7231)

*n*_1_ = number of cases of birth asphyxia in primary hospitals (6433)

*Z*_α/2_ = percentile at (1 – α/2)100% of standard normal for two-sided *t*-test with α level of significance (i.e. Z_0.025_ = 1.96)

*Z*_β_ = percentile at (1 – β) 100% of standard normal for power of test with 1 – β (i.e. *Z*_0.2_ = 0.84)

ρ = intraclass correlation of birth asphyxia with hospitals (pre-setting value)

*n*_*i*_ = sample size average per hospital of birth asphyxia (pre-setting value)

*n*_*j*_ = hospital size estimates

**Fig. 1:**
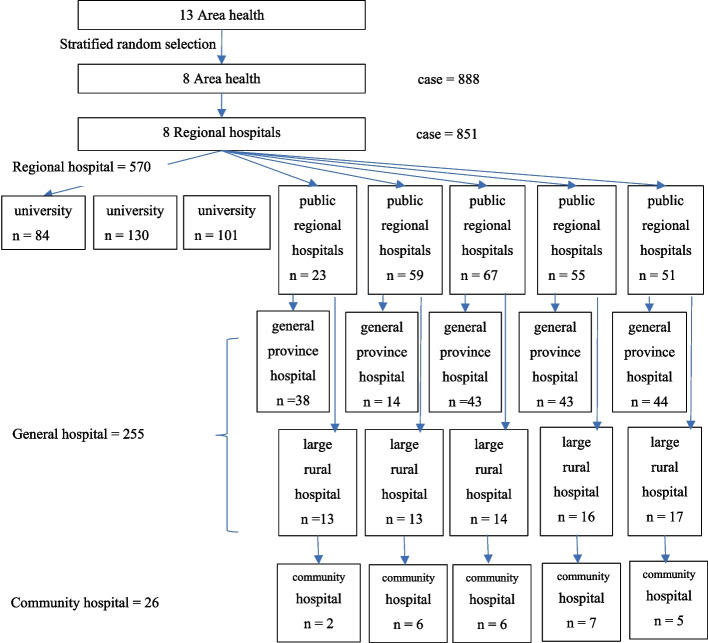
The number and type of hospital settings for data collection

The multilevel study by Ensing et al. [13] was applied to estimate an adequate number of hospitals. According to the formula, the number of research settings (*n*_*j*_) equals 23 hospitals. A Systematic random sampling technique was used to select the research setting (Fig. 1).

## Instruments

The Intrapartum Care Record Form (Supplementary I) collected the intrapartum care quality data via medical chart review. The record form was modified from the Fistula Care Monitoring Tool for Partograph Review of the US Agency for International Development and from the Assessment Tool for the Quality of Hospital Care for Mothers and Newborn Babies of the World Health Organization (2009) [37, 38]. The modified instrument had 12 indicators with 28 items. The score of intrapartum care for pregnant women with reassuring fetuses was 0–30 points and for pregnant women with non-reassuring fetuses was 1–46 points. Therefore, each condition was summed and weighted to 100%. The data were interpreted as four levels: 100%, excellent care quality; 80–99%, good care quality; 50–79%, fair care quality; and 2–49%, poor care quality.

The Asphyxia Risk Factors Record Form (Supplementary II) was used to extract the maternal and fetal risk factor data. The form was modified from the Risk Factors Questionnaire by Aslam et al. (2014) [14]. The modified form comprised 22 Yes/No questions that were separated into three parts: antepartum risk factors (Items 1–9), intrapartum risk factors (Items 10–16) and fetal risk factors (Items 17–22).

The Intrapartum Health Care System Questionnaire (Supplementary III) was modified from the World Health Organization’s Safe Motherhood Needs Assessment Instrument, version 1.1 [39]. The modified questionnaire contained 12 items to collect health services data, the number of pregnant women served by this facility, deliveries per year, high-risk pregnancy, maternity beds, intrapartum healthcare providers and nurse-midwife training. The four additional items include nurse allocation, nurse experience, nurse work-hours and number of cases of birth asphyxia. In addition, all instruments were tested for content validity regarding linguistics, objectivity and comprehensiveness.

## Statistical analysis

Data analysis was conducted using the SPSS statistical software package (version 23). Frequencies and percentages were used to describe the maternal–fetal factors and health service structure. The intrapartum care quality data were calculated for frequency and rate. The intra-class correlation coefficient (ICC) tested for intrapartum health service level variance had a value of > 0.1 [35].

Based on the results of the ICC tests for this study, the ICC value was < 0.1, which indicates that there were no variations of hospital level. Nevertheless, the variations of hospital levels were already explained as the independent variables of research. Therefore, multivariable logistic regression analysis was used to estimate the odds ratio (OR) and 95% confidence interval (95% CI) for predictive factors associated with birth asphyxia. All independent variables were analysed using a univariable model to select the independent variables (presented as *p* < 0.05) with the entering method. Furthermore, model testing for predictive factors was performed by considering the assumption for statistics using multilevel logistic regression analysis. The two required assumptions were that there was no multicollinearity for each independent variable and that the variance–covariance matrices were equal [35].

Because all variables were coded on a dichotomous scale, there was no necessity to test with the two required assumptions. Thus, it was appropriate to analyse the multivariable logistic regression analysis.

## Results

Overall, 23 hospitals delivered health services to 72,005 pregnant women (range 500–9117) during October 2017 to September 2018. The total rate of birth asphyxia was 38.19 per 1000 live births. The total rates of birth asphyxia in advanced hospitals (median = 52.91, range = 17.31–75.24/1000 live births) and university hospitals (median = 30.72, range = 19.40–99.14/1000 live births) were higher than in secondary hospitals (median = 18.87, range = 5.13–51.93/1000 live births). Furthermore, university hospitals had the highest rate of severe birth asphyxia (median = 3.41, range = 0.39–22.44/1000 live births), followed by advanced hospitals (median = 2.29, range = 0–3.39/1000 live birth). The highest number of non-delivered high-risk pregnancies was found in university hospitals (median = 21.93%, range = 1.23–55.70%). Furthermore, the caesarean section rate in university hospitals was the highest (median = 47.28%, range = 7.26–72.70%), followed by advanced hospitals (median = 44.02%, range = 6.03–41.56%; Table 1).

**Table 1:** Number of intrapartum women, type of delivery and newborn health in the three levels of hospital

**Variable**	**Hospital level **			
	**University (** ***n =*** ** 3)**	**Advanced (** ***n =*** ** 5)**	**Secondary (** ***n =*** ** 15)**	**Total (** ***n =*** ** 23)**
**Total number of intrapartum women**				
- Total	16,348 (22.77%)	24,447 (33.95%)	31,210 (43.34%)	**72,005**
- Range	1468 (8.98%)–9117 (55.77%)	2979 (12.19%)–6969 (28.51%)	500 (1.60%)–4010 (12.85%)	500 (0.69%)–9117 (12.66%)
**Non-deliveries with high-risk conditions**				
- Total	5228 (31.98%)	3,534 (14.46%)	5888 (18.87%)	**14,650 (20.35%)**
- Median	21.94%	12.44%	15.27%	14.34%
- Range	18 (1.23%)–3210 (55.70%)	50 (1.22%)–1678 (56.33%)	0–1354 (59.94%)	0–3210 (54.52%)
**Deliveries**				
- Total	11,120 (19.39%)	20,913 (36.46%)	25,322 (44.15%)	**57,355 (79.65%)**
- Median	84.73%	87.56%	78.06%	85.65%
- Range	1013 (13.04%)–2728 (64.00%)	1301 (6.22%)–6102 (29.18%)	494 (1.95%)–3296 (13.26%)	375 (1.20%) 4388 (14.05%)
**Type of Delivery**				
**- Vaginal birth**				
- Total	5097 (16.32%)	12,295 (39.37%)	13,840 (44.34%)	31,232 (54.45%)
- Median	52.72%	55.99%	60.78%	58.81%
- Range	1013 (19.87%)–2738 (53.72%)	781 (6.35%)–4388 (35.69%)	375 (0%)–1733 (12.52%)	375 (0%)–4388 (59.94%)
**- Caesarean birth**				
- Total	6023 (23.06%)	8618 (32.09%)	11,482 (43.95%)	26,123 (45.55%)
- Median	47.28%	44.01%	39.22%	41.19%
- Range	437 (7.26%)–4379 (72.70%)	520 (6.03%)–3582 (41.56%)	102 (0.83%)–1988 (17.31%)	102 (0.39%)–4379 (16.76%)
**Newborn Health Outcomes**				
**- Live birth**				
- Total	12,404 (19.81%)	22,742 (36.33%)	27,459 (43.86%)	62,605
- Range	1465 (11.81%)–7681 (61.92%)	3004 (13.21%)–6067 (26.68%)	390 (1.42%)–3445 (12.55%)	390 (0.62%)–7681 (12.27%)
**- Asphyxia /1000 live births (1st min)**				
- Apgar ≤ 7				
- Total	517 (41.6/1000)	1188 (52.24/1000)	686 (24.98/1000)	2391 (38.19/1000)
- Median	30.72/1000	52.91/1000	18.87/1000	22.13 1000
- Range	(19.40–99.14/1000)	(17.31–75.24/1000)	(5.13–51.93/1000)	(0.84–16.69/1000)
- Apgar ≤ 3				
- Total	81 (6.53/1000)	42 (1.85/1000)	29 (1.06/1000)	152 (2.43/1000)
- Median	3.41/1000	2.29/1000	1.15/1000	1.23/1000
- Range	(0.39–22.44/1000)	(0–3.39/1000)	(0–2.56/1000)	(0–1.17/1000)

Thailand's tertiary level comprised advanced and university hospitals, which can care for more than 500 beds. The secondary level included general and large/middle community hospitals, which can care for more than 90 beds

Among the 23 hospitals, 108 obstetricians, 100 obstetric residents/general physicians and 325 nurse-midwives worked in the intrapartum units. Obstetricians were allocated to all hospitals. The university and advanced hospitals had one or two residents/general physicians assigned to the intrapartum care unit. Of the 325 nurse-midwives, 285 were registered nurse-midwives, 30 received four-month midwifery specialist programme training and 10 were advanced practice nurses. The highest ratio of obstetricians to intrapartum women was found in the university hospitals (1:1440.75; range 209.71–1519.50) and the lowest in the secondary hospitals (1:537.00; range 277.86–1468.00). The nurse-to-client ratios in the university hospitals (median = 260.49, range = 97.87–274.43) and the advanced hospitals (median = 290.38, range = 257.29–383.75) were also higher than in the secondary hospitals (median = 184.46, range = 71.43–300.00). Of the 325 nurse-midwives, 44.62% had worked for ≥ 10 years. The mean nurse-midwife work-hours per week were 46.38 ± 6.78 h. Furthermore, the study found that nurse-midwives in advanced and secondary hospitals work for > 40 h per week (mean = 47.90 ± 4.48 and 47.58 ± 6.90, respectively). The study found that the team nursing care model was the most common (Table 3). Advanced hospitals had the least adequately allocated nurse-midwives for all work shifts (20% in day, 20% in afternoon, 60% at night). Nurse-midwives in all university and advanced hospitals received training on partograph and cardiotocograph monitoring. Furthermore, all nurse-midwives in university hospitals received training in neonatal resuscitation (Table 2).

**Table 2:** The intrapartum health service variables classified by hospital level

**Provider variables **	**Hospital level **			
	**University (** ***n *** **= 3) **	**Advanced (** ***n*** ** = 5) **	**Secondary (** ***n *** **= 15) **	**Total (** ***n *** **= 23) **
- Obstetricians	17	32	59	108
- Resident/general physicians	72	28	-	100
- Nurse-widwives	71	81	173	325
Registered nurse	67	72	146	285
Specialized nurse	3	7	20	30
Advanced practice nurse	1	2	7	10
Nurse-midwife experience				
≤ 3 years	24 (33.80%)	17 (20.90%)	41 (23.70%)	82 (25.23%)
4–9 years	15 (21.13%)	25 (30.86%)	58 (33.53%)	98 (30.15%)
≥ 10 years	32 (45.07%)	39 (48.15%)	74 (42.77%)	145 (44.62%)
** Obstetrician-to-client ratio **				
- Median	1440.75	1023.33	537.00	551.60
- Range	209.71–1519.50	400.22–1189.25	277.86–1468.00	209.71–1519.50
** Nurse-to-client ratio **				
- Median	260.49	290.38	184.46	209.75
- Range	97.87–274.43	257.29–383.75	71.43–300.00	71.43–383.75
** Average nurse work-hours **(hours/week)				
- Mean ± SD	37.8 ± 2.57	47.90 ± 4.48	47.58 ± 6.90	46.38 ± 6.78
- Range	35.00–40.00	42.00–54.00	36.75–56.00	35.00–56.00
** Nursing care model **				
Team	2 (66.67%)	3 (60.00%)	9 (60.00%)	14 (60.87%)
Primary	1 (33.33%)	1 (20.00%)	4 (26.67%)	6 (26.09%)
Combined team and primary	–	1 (20.00%)	2 (13.33%)	3 (13.04%)
**Appropriate nurse staff allocation**/**shift **				
Day (08:00 am–04:00 pm)	2 (66.67%)	1 (20.00%)	14 (93.33%)	17 (73.91%)
Afternoon (04:00 pm–12:00 am)	2 (66.67%)	1 (20.00%)	12 (80.00%)	15 (65.22%)
Night (12:00 am–08:00 am)	2 (66.67%)	3 (60.00%)	15 (100%)	20 (86.96%)
**In-house training programme **				
Partograph use	3 (100%)	5 (100%)	10 (6.67%)	18 (78.26%)
Cardiotocograph monitoring	3 (100%)	5 (100%)	11 (73.33%)	19 (82.61%)
Obstetric emergency management	2 (66.67%)	4 (80.00%)	10 (66.67%)	16 (69.57%)
Neonatal resuscitation	3 (100%)	4 (80.00%)	14 (93.33%)	21 (91.30%)

Appropriate nurse staff allocation based on recommendations of the Thai Nursing Council (nurse-to-intrapartum women ratio of 1:2) (Nursing and Midwifery Council, 2008) and the Association of Women’s Health Obstetric and Neonatal Nurses (women with medical or obstetric complications during labour ratio of 1:1) (Association of Women’s Health Obstetric and Neonatal Nurses, 2010)

Table 3 The quality of intrapartum care provided to 4256 intrapartum women. The study found that 3434 (83.08%) received good quality care. The highest quality was found in university hospitals (93.94%) and the lowest in advanced hospitals (83.04%). Regarding the three main intrapartum care procedures (i.e. careful monitoring, appropriate intervention and activated team alert), secondary hospitals had the most careful monitoring (84.30%), such as partograph plot during active labour (35.89%), cervix dilation observed every 4 h during active labour (37.65%) and descending fetal head observation during active labour (35.12%). University hospitals provided the highest appropriate intervention (32.60%) compared to advanced and secondary hospitals, such as encouraging pregnant women to change their position (64.16%), oxygen management (61.50%), reduced oxytocin (57.05%) and IV fluid loading (61.36%) during non-reassuring fetal status. Regarding team activation, the study found that advanced hospitals were the lowest (44.8%), with a non-reassuring fetal response of 17.49% and a severe non-reassuring fetal termination in 30 min of 5.26%.

**Table 3:** Quality of intrapartum care classified by hospital level

**Intrapartum care**	**University**		**Advanced**		**Secondary**		**Total**	
	***n***	**%**	***n***	**%**	***n***	**%**	***n***	**%**
**Quality of intrapartum care**	(*n =* 1581)		(*n =* 1254)		(*n =* 1421)		(*n =* 4256)	
Mean	93.94 ± 7.24		83.04 ± 14.56		86.07 ± 9.40		88.10±11.53	
**Care quality level**	(*n =* 1581)		(*n =*1254)		(*n =* 1421)		(*n =* 4256)	
Fair (< 80%)	95	6.00	394	31.40	333	23.40	822	19.30
Good (> 80%)	1486	94.00	860	68.60	1088	76.60	3434	80.70
**- Careful monitoring**	(*n =* 1581)		(*n =* 1254)		(*n =* 1421)		(*n =* 4256)	
No	377	23.80	286	22.80	223	15.70	886	20.80
Yes	1204	76.20	968	77.20	1198	84.30	3370	79.20
Partograph use (*n =* 3563)								
No	15	2.55	287	48.81	286	48.64	588	16.50
Yes	1011	33.98	925	31.09	1039	34.92	2975	83.50
Cervical dilatation screening (*n =* 4250)								
No	62	25.41	152	62.3	30	12.30	244	5.74
Yes	1453	36.27	1175	29.33	1378	34.40	4006	94.26
Partograph plot during active labour (*n =* 3397)								
No	8	2.01	244	61.15	147	36.84	399	11.75
Yes	1010	33.69	912	30.42	1076	35.89	2998	88.25
Cervix dilation observed every 4 h during active labour (*n =* 3507)								
No	218	41.76	143	27.39	161	30.84	522	14.88
Yes	1049	35.14	812	27.20	1,124	37.65	2985	85.12
Descending fetal head observed during active labour (*n =* 4072)								
No	199	52.93	162	43.09	15	3.99	376	9.23
Yes	1270	34.36	1128	30.52	1298	35.12	3696	90.77
Contractions observed every 30 min during active labour (*n =* 3697)								
No	101	10.43	427	44.11	440	45.45	968	26.18
Yes	1260	46.17	672	24.62	797	29.20	2729	73.82
Amniotic fluid membrane monitoring (*n =* 3998)								
No	8	4.23	144	76.19	37	19.58	189	4.73
Yes	1360	35.70	1131	29.69	1318	34.60	3809	95.27
Amniotic fluid characteristic record (*n =* 3838)								
No	9	6.21	129	88.97	7	4.83	145	3.78
Yes	1356	36.72	1059	28.68	1278	34.61	3693	96.22
FHR monitoring every 30 min during active labour (*n =* 3628)								
No	67	7.25	405	43.83	452	48.92	924	25.47
Yes	1199	44.34	708	26.18	797	29.47	2704	74.53
Blood pressure screening on admission (*n =* 4183)								
No	0	0.00	110	98.21	2	1.79	112	2.68
Yes	1453	35.69	1212	29.77	1406	34.54	4071	97.32
Blood pressure monitoring every 4 h (*n =* 3804)								
No	3	2.38	86	68.25	37	29.37	126	3.31
Yes	1381	37.55	992	26.97	1305	35.48	3678	96.69
Pulse screening on admission (*n =* 4121)								
No	1	0.78	125	96.9	3	2.33	129	3.06
Yes	1420	34.78	1248	30.57	1415	34.66	4083	96.94
Pulse monitoring every 4 h (*n =* 3804)								
No	3	2.38	86	68.25	37	29.37	126	3.31
Yes	1381	37.55	992	26. 97	1305	35.48	3678	96.69
Temperature monitoring every 4 h (*n =* 4210)								
No	7	4.35	124	77.02	30	18.63	161	3.82
Yes	1416	34.97	1248	30.82	1385	34.21	4049	96.18
FHR monitoring in 2^nd^ stage (*n =* 2248)								
No	4	0.47	360	42.2	489	57.33	853	37.94
Yes	457	32.76	490	35.13	448	32.11	1395	62.06
Severe NRFS monitoring every 5 min during 2^nd^ stage (*n =* 771)								
No	117	28.61	216	52.81	76	18.58	409	53.05
Yes	163	45.03	140	38.67	59	16.30	362	46.95
**- Appropriate intervention**	(*n =* 1521)		(*n =* 1254)		(*n =* 1421)		(*n =* 4256)	
No	1006	67.40	1065	84.90	1199	84.40	3330	78.20
Yes	515	32.60	189	15.10	222	15.60	926	21.80
Encourage position change during active labour (*n =* 3645)								
No	551	30.26	545	29.93	725	39.81	1821	49.96
Yes	721	39.53	511	28.02	592	32.46	1824	50.04
Clear bladder during active labour (*n =* 3607)								
No	543	34.90	535	34.38	478	30.72	1556	43.14
Yes	733	35.74	496	24.18	822	40.08	2051	56.86
Uterus stimulated during active labour (*n =* 4021)								
No	739	30.93	727	30.43	923	38.64	2389	59.41
Yes	793	48.59	482	29.53	357	21.88	1632	40.59
Encourage position change during NRFS (*n =* 829)								
No	81	39.71	63	30.88	60	29.41	204	24.61
Yes	401	64.16	104	16.64	120	19.20	625	75.39
Oxygen management during NRFS (*n =* 839)								
No	30	28.85	50	48.08	24	23.08	104	12.40
Yes	452	61.50	127	17.28	156	21.22	735	87.60
Reduced oxytocin during NRFS (*n =* 823)								
No	304	59.49	94	18.4	113	22.11	511	62.09
Yes	178	57.05	67	21.47	67	21.47	312	37.91
Intravenous fluid loading during NRFS (*n =* 845)								
No	220	52.63	111	26.56	87	20.81	418	49.47
Yes	262	61.36	72	16.86	93	21.78	427	50.53
**- Team activated**	(*n =* 332)		(*n =* 1001)		(*n =* 248)		(*n =* 1581)	
No	88	26.5	553	55.2	69	27.8	710	44.9
Yes	244	73.5	448	44.8	179	72.2	871	55.1
For prolonged active labour (*n =* 1338)								
No	48	10.28	345	73.88	74	15.85	467	34.90
Yes	210	24.11	360	41.33	301	34.56	871	65.10
For NRFS (*n =* 831)								
No	19	32.20	34	57.63	6	10.17	59	7.10
Yes	463	59.97	135	17.49	174	22.54	772	92.90
For prolonged ^2nd^ stage (*n =* 181)								
No	53	54.64	28	28.87	16	16.49	97	53.59
Yes	9	10.71	39	46.43	36	42.86	84	46.41
Severe NRFS terminated in 30 min (*n =* 86)								
No	38	56.72	3	4.48	26	38.81	67	77.91
Yes	6	31.58	1	5.26	12	63.16	19	22.09

Totals do not necessarily add up across all variables because of missing data in the medical chart records

*FHR* Fetal heart rate, *NRFS* Non-reassuring fetal status

Table 4 presents the binary logistic regression results for factors contributing to birth asphyxia. The univariablete analysis found factors predicted birth asphyxia such as the quality of intrapartum care, delivery conducted provider, gestational age, parity, birth weight, maternal comorbidity, fetal complication, obstetric emergency, and non-reassuring fetal status. The odd of birth asphyxia was significantly increased 1.45-fold in pregnant women who received good quality care compared to excellent care (95% CI = 1.19–1.77, *p* < 0.001). Furthermore, deliveries conducted by residents or general physicians significantly increased the odds of birth asphyxia to 2.06 times for deliveries by obstetricians (95% CI = 1.68–2.52, *p* < 0.001). As for gestational age, the odd of birth asphyxia increased significantly by 1.69- and 1.53-fold at late-preterm (34^+0^–36^+6^ weeks) and late-term (41^+0^–41^+6^ weeks), respectively, compared to full-term deliveries (37^+0^–40^+6^ weeks): 95% CI = 1.43–2.01 (*p* = 0.000) and 95% CI = 1.08–2.16 (*p* = 0.018), respectively. The odd of birth asphyxia was also significantly higher in the nulliparous group than the multiparous group (OR = 1.25, 95% CI = 1.073–1.467, *p* = 0.004). The odd of birth asphyxia increased significantly by 2.31- and 1.99-fold in low-birthweight newborns (≤ 2500 g) and newborns with macrosomia (≥ 4000 g), respectively, compared to the normal-weight newborns (2501–3999 g): 95% CI = 1.81–2.95 (*p* = 0.000) and 95% CI = 1.32–3.00 (*p* = 0.001), respectively. Furthermore, the odd of birth asphyxia was significantly increased in the maternal–fetal high-risk groups: maternal comorbidity, 1.73-fold (95% CI = 1.47–2.03, *p* < 0.001); fetal complications, 2.21 -fold (95% CI = 1.69–2.88, p < 0.001); and obstetric emergency, 1.87-fold (95% CI = 1.50–2.33, *p* < 0.001). In particular, non-reassuring fetal status significantly increased the odd of birth asphyxia by 3.07-fold (95% CI = 2.59–3.63, *p* < 0.001).

**Table 4:** The Binary Logistic Regression Analysed Factors Contributing to Birth Asphyxia in Thailand, 2016 – 2017

Variable	Asphyxia		No Asphyxia		OR	95% CI		*p*	aOR	95% CI		*p*
	***n***	**%**	***n***	**%**								
**Quality of intrapartum care**	851	20.00	3405	80.00								
- Excellent (100%)	153	17.97	773	22.70	*ref*				*ref*			
- Good (80–99%)	139	16.33	1949	57.24	1.45	1.19	1.77	< *0.001*	1.47	1.17	1.84	*0.001*
- Fair–Low (< 50%)	559	65.69	683	20.06	1.03	0.80	1.32	*0.828*	1.01	0.77	1.34	*0.924*
**Hospital level**												
- University	315	37.02	1266	37.18	*ref*							
- Advanced	298	35.02	1180	34.65	1.015	0.850	1.23	*0.869*				
- Secondary	238	27.97	959	28.16	0.997	0.826	1.19	*0.979*				
**Nursing care model**												
- Team	476	55.90	1963	57.70	*ref*				*ref*			
- Primary	234	27.50	917	26.90	1.052	0.883	1.254	*0.568*	1.58	1.24	2.02	< *0.001*
- Combined	141	16.60	525	15.40	1.108	0.897	1.368	*0.343*	0.74	0.57	0.96	*0.021*
**Specialized nurse**												
- Unavailable	298	35.00	1207	35.40	*ref*				*ref*			
- Available	553	65.00	2198	64.60	1.02	0.87	1.19	*0.814*	1.21	0.96	1.53	*0.106*
**Advanced practice nurse**												
- Unavailable	660	20.00	2645	80.00	*ref*							
- Available	191	20.10	760	79.90	1.01	0.84	1.21	*0.938*				
**Nurse work-hours (hours/week)**												
- ≥ 40 h	667	78.37	2698	79.27	*ref*				*ref*			
- < 40 h	184	21.62	707	20.76	.095	0.79	1.14	*0.582*	0.62	0.50	0.77	< *0.001*
**Delivery conducted by:**												
- Obstetrician	481	58.40	2070	65.40	*ref*				*ref*			
- Nurse-midwives	155	18.80	703	22.20	0.95	0.78	1.16	*0.607*	1.02	0.82	1.27	*0.880*
- Resident or general physician	187	22.70	391	12.40	2.06	1.68	2.52	< *0.001*	1.99	1.58	2.49	< *0.001*
**Gestational group**												
- Term	556	65.50	2584	76.00	*ref*				*ref*			
- Late-preterm(34^+0^–36^+6^ weeks)	248	29.20	680	20.00	1.69	1.43	2.01	< *0.001*	1.63	1.34	1.98	< *0.001*
- Late-term (40^+1^–41^+6^ weeks)	45	5.30	137	4.00	1.53	1.08	2.16	*0.018*	1.61	1.11	2.34	*0.012*
**Parity**												
- Multiparous	550	35.40	2019	40.70	*ref*							
- Nulliparous	301	64.60	1386	59.30	1.25	1.073	1.467	*0.004*				
**Pre-pregnancy BMI**												
- Normal weight	304	42.70	1235	42.00	*ref*							
- Underweight	277	38.90	1289	43.80	0.87	0.73	1.05	*0.140*				
- Overweight	91	12.80	288	9.80	1.28	0.98	1.68	*0.067*				
- Obese	40	5.60	128	4.40	1.27	0.87	1.85	*0.214*				
**Completion of ANC visits**												
- Incomplete	322	40.20	1292	40.80	*ref*							
- Complete	479	59.80	1877	59.20	1.02	0.87	1.20	*0.769*				
**Birth weight**												
- Normal	128	15.00	231	6.80	*ref*				*ref*			
- Low	689	81.00	3096	90.90	2.31	1.81	2.95	< *0.001*	2.29	1.77	2.96	< *0.001*
- Macrosomia	34	4.00	78	2.30	1.99	1.32	3.00	*0.001*	2.14	1.37	3.35	*0.001*
**Maternal and fetal high-risk conditions**												
**Maternal comorbidity**												
- No	562	66.04	2624	77.06	*ref*				*ref*			
- Yes	289	33.96	781	22.94	1.73	1.47	2.03	< *0.001*	1.65	1.35	1.94	< *0.001*
**Intrapartum complications**												
- No	737	86.60	2904	85.30	*ref*							
- Yes	114	13.40	501	14.70	0.90	0.72	1.12	*0.328*				
**Fetal complications**												
- No	760	89.30	3230	94.90	*ref*							
- Yes	91	10.70	175	5.10	2.21	1.69	2.88	< *0.001*				
**Obstetric emergency**												
- No	722	84.80	3108	91.30	*ref*				*ref*			
- Yes	129	15.20	297	8.70	1.87	1.50	2.33	< *0.001*	1.64	1.283	2.11	< *0.001*
**Non-reassuring fetal status**												
- Reassuring	552	64.86	2894	83.98	*ref*				*ref*			
- Non-reassuring	299	35.14	511	14.83	3.07	2.59	3.633	< *0.001*	3.04	2.51	3.69	< *0.001*
									*Nagelkerke R*^*2*^ = *0.140*			

*CTG* Cardiotocography, *ref* reference, *ANC* Antenatal care, *BMI* Body mass index, *aOR* adjusted odds ratio

Table 4 presents the binary logistic regression results for factors contributing to birth asphyxia. The univariable analysis found factors predicted birth asphyxia such as the quality of intrapartum care, delivery conducted provider, gestational age, parity, birth weight, maternal comorbidity, fetal complication, obstetric emergency, and non-reassuring fetal status. The odd of birth asphyxia was significantly increased 1.45-fold in pregnant women who received good quality care compared to excellent care (95% CI = 1.19–1.77, *p* < 0.001). Furthermore, deliveries conducted by residents or general physicians significantly increased the odds of birth asphyxia to 2.06 times for deliveries by obstetricians (95% CI = 1.68–2.52, *p* < 0.001). As for gestational age, the odd of birth asphyxia increased significantly by 1.69- and 1.53-fold at late-preterm (34^+0^–36^+6^ weeks) and late-term (41^+0^–41^+6^ weeks), respectively, compared to full-term deliveries (37^+0^–40^+6^ weeks): 95% CI = 1.43–2.01 (*p* = 0.000) and 95% CI = 1.08–2.16 (*p* = 0.018), respectively. Birth asphyxia was also significantly higher in the nulliparous group than the multiparous group (OR = 1.25, 95% CI = 1.073–1.467, *p* = 0.004). The odd of birth asphyxia increased significantly by 2.31- and 1.99-fold in low-birthweight newborns (≤ 2500 g) and newborns with macrosomia (≥ 4000 g), respectively, compared to normal-weight newborns (2501–3999 g): 95% CI = 1.81–2.95 (*p* = 0.000) and 95% CI = 1.32–3.00 (*p* = 0.001), respectively. Furthermore, odd of birth asphyxia was significantly increased in the maternal–fetal high-risk groups: maternal comorbidity, 1.73-fold (95% CI = 1.47–2.03, *p* < 0.001); fetal complications, 2.21 -fold (95% CI = 1.69–2.88, *p* < 0.001); and obstetric emergency, 1.87-fold (95% CI = 1.50–2.33, *p* < 0.001). In particular, non-reassuring fetal status significantly increased the odd of birth asphyxia by 3.04-fold (95% CI = 2.51–3.69, *p* < 0.001).

However, the multivariable logistic regression results for the factors contributing to birth asphyxia. According to the chi-square statistic model, the overall model is significant at *p* < 0.001. The Nagelkerke R^2^ value of 0.140 suggests that approximately 14% of the variation in the response variable is explained by the predictors included in the logistic regression model. The analysis indicated that the odd of birth asphyxia was significantly higher with good care quality compared to excellent care quality (aOR = 1.47, 95% CI = 1.17–1.84, *p* = 0.001). Birth asphyxia was significantly reduced by 38% for nurse work-hours of < 40 h per week (aOR = 0.62, 95% CI = 0.50–0.77, *p* < 0.001). Furthermore, the results show that birth asphyxia was significantly reduced by 26% when the combined nursing care model (primary and team) was applied in hospitals compared to the team nursing care model (aOR = 0.74, 95% CI = 0.57–0.96, *p* = 0.021). In contrast, the primary care model had significantly higher the odd of birth asphyxia than the team nursing care model (aOR = 1.58, 95% CI = 1.24–2.02, *p* ≤ 0.001). The odd of birth asphyxia was significantly increased when the newborn was delivered by a resident or general physician rather than by an obstetrician (aOR = 1.99, 95% CI = 1.58–2.49, *p* < 0.001). Moreover, delivery in the late-preterm and late-term groups significantly increased odd of birth asphyxia compared to the full-term pregnancy group (aOR = 1.63, 95% CI = 1.34–1.98, *p* < 0.001; aOR = 1.61, 95% CI = 1.11–2.34, *p* = 0.012). Pregnancies with comorbidity, obstetric emergency, non-reassuring fetal status, low birthweight or macrosomia significantly increased odd of birth asphyxia (aOR = 1.62, 95% CI = 1.35–1.94, *p* < 0.001; aOR = 1.64, 95% CI = 1.29–2.11, *p* < 0.001; aOR = 3.04, 95% CI = 2.50–3.69, *p* < 0.001; aOR = 2.29, 95% CI = 1.77–2.96, *p* < 0.001; aOR = 2.14, 95% CI = 1.37–3.35, *p* = 0.001)*.*

## Discussion

This case–control study aimed to determine the maternal–fetal and health service factors contributing to birth asphyxia. Among the 4256 samples, the average rate of birth asphyxia was 38.19 per 1000 live births. Although advanced hospitals had the highest rate of birth asphyxia (52.24 per 1000 live births), university hospitals had the highest rate of severe birth asphyxia (6.53 per 1000 live births), possibly due to the number of high-risk deliveries referred from other facilities.

Multivariable logistic regression analysis found significant increases the odds of birth asphyxia in late-preterm, late-term, low-birth weight, and macrosomia fetuses. This result was supported by previous studies finding that neonates born at 34^+0^–36^+6^ weeks and of low birthweight are associated with birth asphyxia. The immature pulmonary function may make it difficult for the fetus to maintain breathing after birth. Likewise, the placental deterioration in post-term neonates increases the likelihood of birth asphyxia more than in pregnancies delivered at term. Therefore, non-spontaneous late-term deliveries should be avoided in order to reduce birth asphyxia. Antenatal surveillance is recommended at 40^+0^–41^+6^ weeks, beginning twice weekly with the biophysical profile or non-stress testing plus amniotic fluid index measure, along with induction at 41^+0^ weeks’ gestation [40–43].

Furthermore, the study found that birth asphyxia increases significantly in pregnant women with comorbidities and obstetric emergency conditions. Maternal complications such as gestational hypertension and diabetes may cause utero-placental insufficiency, resulting in reduced blood flow and loss of placental integrity that predisposes the fetus to intrauterine hypoxia [44–46]. An obstetric emergency during the intrapartum period significantly increases the likelihood of birth asphyxia. Maternal–fetal circulatory problems could also occur in placenta abruption, cord prolapse, shoulder dystocia and non-reassuring fetus [47–50]. Therefore, intrapartum care by carefully screening, close monitoring, timely detection and appropriate response from a multidisciplinary team are needed to resolve the problem.

The research findings revealed that an excellent level of intrapartum care quality reduces birth asphyxia. Careful monitoring, such as partograph use for monitoring the descent of the fetal head to identify the cephalo-pelvic disproportion promptly, can effectively reduce birth asphyxia. Continuous fetal heart rate monitoring in non-reassuring fetal conditions can shorten the decision-to-delivery time and reduce the severity of hypoxic-ischaemic encephalopathy in newborns [51, 52]. Appropriate interventions, such as placing intrapartum women in a side-lying position with the head elevated, can significantly reduce the risk of birth asphyxia. In a severe non-reassuring fetus, the appropriate intrauterine resuscitation reduced fetal hypoxia. Activating an alert team to respond, such as obstetricians, paediatricians, anaesthetists and specialized or experienced nurses, could reduce birth asphyxia [23–28, 34]. Furthermore, the study found that caesarean delivery reduced birth asphyxia significantly. Under high-risk pregnancy conditions or obstetric emergencies, caesarean delivery may be required to save the mother’s and the baby's lives and prevent unexpected adverse outcomes.

Furthermore, the research found that the combined primary and team nursing care model significantly lowered birth asphyxia compared to the team nursing care model. The result is different from the previous study, which found that the one-on-one care or primary/total patient care model can ensure that pregnant women received closer and more continuous care and had more desirable outcomes than other nursing models [53]. However, the primary care model had a limitation for nurses with less experience because it requires that they work and make decisions alone [54]. Therefore, in a nurse staff shortage situation such as that in Thailand, primary nursing care combined with the team nursing care model may be reasonable for intrapartum care. Furthermore, training to improve competency and confidence in providing intrapartum care, including electronic fetal monitoring, emergency obstetric management and a neonatal resuscitation training programme to improve neonatal outcomes, should be provided to fewer experienced nurse-midwives in labour and delivery units [23, 55].

Lastly, the study found that newborns delivered by a resident or general physician had a higher risk of birth asphyxia. This may be because the general physicians or residents may not be trained to deal with complicated patients referred to the advanced and university-level hospitals. The high workload condition made it difficult to improve the quality of nursing care, therefore medical work experience requires the obstetrician to be closely supervised in order to improve client health outcomes [56].

## Implications for practice

Pregnant women with comorbidity and late-preterm, late-term pregnancies, low-birth weigh, and macrosomia need close monitoring to prevent birth asphyxia. In addition, an appropriate number of nurse-midwives allocated in the advanced hospitals is needed to resolve the high workload, transferring non-intrapartum pregnant women with high-risk conditions to the appropriate unit to be managed by a specialized multidisciplinary team. Furthermore, the intrapartum nursing care model driven by applying the primary care model combined with the team model is feasible for providing good quality care.

## Limitations

The retrospective case–control study was designed for data extraction from medical chart reviews, therefore unreported healthcare activity was not available to clarify the quality of intrapartum care.

To ensure that birth asphyxia did not develop from immature fetal lungs or placental insufficiency, the research samples did not include very preterm and or post-term fetuses in the selection criteria and therefore such fetuses were not considered as factors affecting birth asphyxia in this study.

## Conclusion

Birth asphyxia in Thailand is a serious problem that requires more attention. An excellent level of intrapartum care quality is required to reduce the birth asphyxia rate, carefully evaluating and monitoring pregnant women with comorbidity, late-preterm and late-term pregnancies and newborns with low birthweight or macrosomia. The primary care model combined with the team nursing care model is the alternative strategy to improve the quality of care in the intrapartum unit. In addition, obstetric emergencies need early detection and appropriate intervention by a specialized care team to enhance staff allocation and reduce the nurse work-hours per week .

### Supplementary Information

**Additional file 1.** Intrapartum Care Record Form.

**Additional file 2.** Asphyxia Risk Factors Record Form.

**Additional file 3.** Intrapartum Health Care System Questionnaire.

## Data Availability

The datasets used and/or analysed during the current study are available from the corresponding author on reasonable request.

## References

[1] Ahearne CE, Boylan GB, Murray DM (2016). Short and long term prognosis in perinatal asphyxia: An update. World J. Clin. Pediatr..

[2] Goldenberg RL, Harrison MS, McClure EM (2016). Stillbirths: The hidden birth asphyxia - US and global perspectives. Clin. Perinatol..

[3] Pappas A, Korzeniewski SJ (2016). Long-term cognitive outcomes of birth asphyxia and the contribution of identified perinatal asphyxia to cerebral palsy. Clin. Perinatol..

[4] Garfinkle J, Wintermark P, Shevell MI, Oskoui M (2016). Cerebral palsy after neonatal encephalopathy: Do neonates with suspected asphyxia have worse outcomes?. Dev. Med. Child Neurol..

[5] Sendeku FW, Azeze GG, Fenta SL (2020). Perinatal asphyxia and its associated factors in Ethiopia: A systematic review and meta-analysis. BMC Pediatr..

[6] Abdo RA, Halil HM, Kebede BA, Anshebo AA, Gejo NG (2019). Prevalence and contributing factors of birth asphyxia among the neonates delivered at Nigist Eleni Mohammed memorial teaching hospital, Southern Ethiopia: A cross-sectional study. BMC Pregnancy Childbirth.

[7] Workineh Y, Semachew A, Ayalew E, Animaw W, Tirfie M, Birhanu M (2020). Prevalence of perinatal asphyxia in East and Central Africa: Systematic review and meta-analysis. Heliyon.

[8] Ministry of Public Health (Thailand), Department of Health. Birth Asphyxia Rate 2021. Department of Health Dashboard. 2021. Available from: https://dashboard.anamai.moph.go.th/dashboard/birthasphyxia/index?year=2021.

[9] Greco P, Nencini G, Piva I, Scioscia M, Volta CA, Spadaro S, Nappi L (2020). Pathophysiology of hypoxic-ischemic encephalopathy: A review of the past and a view on the future. Acta Neurol. Belg..

[10] Strategy and Planning Division. Health Statistic 2017. Strategy and Planning Division, Ministry of Public Health, Thailand. 2018. Available from: https://bps.moph.go.th/new_bps/sites/default/files/stratistics60.pdf.

[11] Gillam-Krakauer M, Gowen CW (2020). Birth Asphyxia.

[12] Ayuk Widiani N, Yuli Kurniati D, Windiani IT (2016). Maternal and infant risk factors on the incidence of neonatal asphyxia in Bali: A case-control study. Publ. Health Prev. Med. Arch..

[13] Ensing S, Abu-Hanna A, Schaaf JM, Mol BWJ, Ravelli ACJ (2015). Trends in birth asphyxia, obstetric interventions and perinatal mortality among term singletons: A nationwide cohort study. J. Matern. Fetal Neonatal Med..

[14] Aslam HM, Saleem S, Afzal R, Iqbal U, Saleem S, Shaikh MWA, Shahid N (2014). Risk factors of birth asphyxia. Ital. J. Pediatr..

[15] Tu JH, Profit J, Melsop K, Brown T, Davis A, Main E, Lee HC (2017). Relationship of hospital staff coverage and delivery room resuscitation practices to birth asphyxia. Am. J. Perinatol..

[16] Morioka N, Tomio J, Seto T, Kobayashi Y (2017). The association between higher nurse staffing standards in the fee schedules and the geographic distribution of hospital nurses: A cross-sectional study using nationwide administrative data. BMC Nursing.

[17] Liu X, Zheng J, Liu K, Baggs JG, Liu J, Wu Y, You L (2018). Hospital nursing organisational factors, nursing care left undone, and nurse burnout as predictors of patient safety: A structural equation modeling analysis. Int. J. Nurs. Stud..

[18] Amiri A, Vehviläinen-Julkunen K, Solankallio-Vahteri T, Tuomi S (2020). Impact of nurse staffing on reducing infant, neonatal and perinatal mortality rates: Evidence from panel data analysis in 35 OECD countries. Int. J. Nurs. Sci..

[19] Stimpfel AW, Aiken LH (2013). Hospital staff nurses' shift length associated with safety and quality of care. J. Nurs. Care Qual..

[20] Sharma S, Rani R (2020). Nurse-to-patient ratio and nurse staffing norms for hospitals in India: A critical analysis of national benchmarks. J. Family Med. Prim. Care.

[21] Mildenberger C, Ellis C, Lee K (2017). Neonatal resuscitation training for midwives in Uganda: Strengthening skill and knowledge retention. Midwifery.

[22] Ljungblad LW, Sandvik SO, Lyberg A (2019). The impact of skilled birth attendants trained on newborn resuscitation in Tanzania: A literature review. Int. J. Afr. Nurs. Sci..

[23] Johnson MJ (2020). Intrauterine fetal resuscitation: A midwife’s role. Glob. J. Res. Anal..

[24] Housseine N, Punt MC, Mohamed AG, Said SM, Maaløe N, Zuithoff NPA, Rijken MJ (2020). Quality of intrapartum care: Direct observations in a low-resource tertiary hospital. Reprod. Health.

[25] Kc A, Wrammert J, Clark RB, Ewald U, Målqvist M (2016). Inadequate fetal heart rate monitoring and poor use of partogram associated with intrapartum stillbirth: A case-referent study in Nepal. BMC Pregnancy Childbirth.

[26] Masereka EM, Naturinda A, Tumusiime A, Munguiko C (2020). Implementation of the Perinatal Death Surveillance and Response guidelines: Lessons from annual health system strengthening interventions in the Rwenzori Sub-Region Western Uganda. . Nurs. Open.

[27] Ibrahim Gouda AM, Hassan Khedr NF (2018). Effect of left lateral position on intrauterine fetal resuscitation among pregnant women with reduced fetal movements. Int. J. Nurs. Didact..

[28] Prescott KM, Semroc B (2019). Creating a culture of excellence around fetal heart rate tracings through listening and collaboration. J. Obstet. Gynecol. Neonatal Nurs..

[29] Havaei F, MacPhee M, Dahinten VS (2019). The effect of nursing care delivery models on quality and safety outcomes of care: A cross-sectional survey study of medical-surgical nurses. J. Adv. Nurs..

[30] Sosa GA, Crozier KE, Stockl A (2018). Midwifery one-to-one support in labour: More than a ratio. Midwifery.

[31] Gidaszewski B, Khajehei M, Gibbs E, Chua SC (2019). Comparison of the effect of caseload midwifery program and standard midwifery-led care on primiparous birth outcomes: A retrospective cohort matching study. Midwifery.

[32] Dal Molin A, Gatta C, Boggio Gilot C, Ferrua R, Cena T, Manthey M, Croso A (2018). The impact of primary nursing care pattern: Results from a before–after study. J. Clin. Nurs..

[33] Wan H, Hu S, Thobaben M, Hou Y, Yin T (2011). Continuous primary nursing care increases satisfaction with nursing care and reduces postpartum problems for hospitalised pregnant women. Contemp. Nurse.

[34] Berazategui JP, Aguilar A, Escobedo M, Dannaway D, Guinsburg R, de Almeida MFB, Szyld E (2017). Risk factors for advanced resuscitation in term and near-term infants: A case–control study. Arch. Dis. Child. Fetal Neonatal Ed..

[35] Plichta SB, Kelvin EA (2001). Munro's Statistical Methods for Health Care Research.

[36] Cohen J (1977). Statistical Power Analysis for the Behavioural Sciences.

[37] USAID (2014). Fistula Care and Engender Health. https://www.fistulacare.org. Accessed 12 May 2021.

[38] World Health Organization. Making Pregnancy Safer Assessment Tool for the Quality of Hospital Care for Mothers and Newborn Babies. 2009. https://apps.who.int/iris/handle/10665/107968. Accessed 12 May 2021.

[39] World Health Organization. Safe Motherhood Needs Assessment Version 1.1 – 2001 (Revised edition). Sexual and Reproductive Health. 2021. Retrieved from: https://www.who.int/reproductivehealth/publications/maternal_perinatal_health/rht_msm_96_18/en/. Accessed 12 May 2021.

[40] Ranjbar A, Mehrnoush V, Darsareh F, Pariafsay F, Shirzadfardjahromi M, Shekari M, Darsareh Sr F. The Incidence and Outcomes of Late-Term Pregnancy. Cureus. 2023;15(1). 10.7759/cureus.33550 PMC9907390 36779141

[41] Murzakanova G, Räisänen S, Jacobsen AF, Sole KB, Bjarkø L, Laine K. Adverse perinatal outcomes in 665,244 term and post-term deliveries—a Norwegian population-based study. Euro J Obstet Gynecol Reprod Biol. 2020;247:212–8. 10.1016/j.ejogrb.2020.02.028 32146227

[42] Kortekaas JC, Bruinsma A, Keulen J, Vandenbussche F, van Dillen J, de Miranda E. Management of late-term pregnancy in midwifery- and obstetrician-led care. BMC Pregnancy Childbirth. 2019;19(1):181. https://doi-org.ejournal.mahidol.ac.th/10.1186/s12884-019-2294-7. 10.1186/s12884-019-2294-7 PMC6532173 31117985

[43] Maoz O, Wainstock T, Sheiner E, Walfisch A. Immediate perinatal outcomes of postterm deliveries. J Maternal Fetal Neonatal Med. 2018;32(11):1847–52. 10.1080/14767058.2017.1420773 29301466

[44] Newman C, Egan AM, Ahern T, Al-Kiyumi M, Balan G, Brassill MJ, Brosnan E, Carmody L, Clarke H, Coogan Kell, C, Culliney L, Davern R, Durkan M, Fenlon M, Ferry P, Hanlon G, Higgins T, Hoashi S, Khamis A, Kinsley B, Dunne FP. Diabetes care and pregnancy outcomes for women with pregestational diabetes in Ireland. Diabetes Res Clin Pract. 2021;173:108685. 10.1016/j.diabres.2021.108685. 10.1016/j.diabres.2021.108685 33548336

[45] Agrawal A, nger NK. Hypertension during pregnancy. Curr Hypertens Rep. 2020;22(9):64. 10.1007/s11906-020-01070-0. 10.1007/s11906-020-01070-0 32852628

[46] Smith C, Teng F, Branch E, Chu S, Joseph KS. Maternal and perinatal morbidity and mortality associated with anemia in pregnancy. Obstet. Gynecol. 2019;134(6):1234–44. 10.1097/AOG.0000000000003557. 10.1097/AOG.0000000000003557 PMC6882541 31764734

[47] Downes KL, Grantz KL, Shenassa ED. Maternal, labor, delivery, and perinatal outcomes associated with placental abruption: A systematic review. Am J Perinatol. 2017;34(10): 935–57. 10.1055/s-0037-1599149. 10.1055/s-0037-1599149 PMC5683164 28329897

[48] Li Y, Tian Y, Liu N, Chen Y, Wu F. Analysis of 62 placental abruption cases: Risk factors and clinical outcomes. Taiwan J Obstet Gynecol. 2019;58(2):223–6. 10.1016/j.tjog.2019.01.010. 10.1016/j.tjog.2019.01.010 30910143

[49] Adeniran AS, Imhoagene A, Ezeoke GG. Presentation and perinatal outcome following umbilical cord prolapse in Ilorin. J Trop Med. 2017;19(1):31–5. 10.4103/jomt.jomt_39_16.

[50] Cesari E, Ghirardello S, Brembilla G, Svelato A, Ragusa A. Clinical features of a fatal shoulder dystocia: The hypovolemic shock hypothesis, *Med Hypotheses.* 2018;118:139–41. https://doi.org/30037602. 10.1016/j.mehy.2018.07.006 30037602

[51] Salma U, Jabeen M, Shimul S, Akhter D. Analysis of cardiotocography findings in pregnancy with less fetal movement and its association with perinatal outcome. Med Today. 2018;30(1):19-22. https://doi.org/58484206.

[52] Kitaw TM, Limenh SK, Chekole FA, Getie SA, Gemeda BN, Engda AS. Decision to delivery interval and associated factors for emergency cesarean section: A cross-sectional study. BMC Pregnancy Childbirth. 2021;21:224. https://doi.org/https://doi-org.ejournal.mahidol.ac.th/10.1186/s12884-021-03706-8. 10.1186/s12884-021-03706-8 PMC7981954 33743626

[53] Fernandez R, Johnson M, Tran DT, Miranda C. Models of care in nursing: A systematic review. Int J Evid‐Based Healthc. 2012;10(4):324–37. https://doi.org/https://scholar-google-com.ejournal.mahidol.ac.th/scholar?hl=th&as_sdt=0%2C5&q=Models+of+care+in+nursing%3A+A+systematic+review.&btnG=. 10.1111/j.1744-1609.2012.00287.x 23173657

[54] Fairbrother G, Chiarella M, Braithwaite J. Models of care choices in today's nursing workplace: Where does team nursing sit? Aust Health Rev. 2015;39(5):489–93. https://doi.org/https://scholar-google-com.ejournal.mahidol.ac.th/scholar?hl=th&as_sdt=0%2C5&q=Models+of+care+choices+in+today%27s+nursing+workplace%3A+Where+does+team+nursing+sit%3F+&btnG=. 10.1071/AH14091 26143068

[55] Desta M, Akalu TY, Alamneh YM, Talie A, Alemu AA, Tessema Z, Getaneh T. Perinatal mortality and its association with antenatal care visit, maternal tetanus toxoid immunization and partograph utilization in Ethiopia: a meta-analysis. Sci Rep. 2021;11(1):19641. https://doi.org/https://scholar-google-com.ejournal.mahidol.ac.th/scholar?hl=th&as_sdt=0%2C5&q=Perinatal+mortality+and+its+association+with+antenatal+care+visit%2C+maternal+tetanus+toxoid+immunization+and+partograph+utilization+in+Ethiopia%3A+a+meta-analysis&btnG=. 10.1038/s41598-021-98996-5 PMC8490438 34608180

[56] Bardos J, Loudon H, Rekawek P, Friedman F, Brodman M, Fox NS. Association between senior obstetrician supervision of resident deliveries and mode of delivery. Obstet. Gynecol. 2017;129(3):486–90. https://doi.org/https://journals-lww-com.ejournal.mahidol.ac.th/greenjournal/fulltext/2017/03000/Association_Between_Senior_Obstetrician.12.aspx. 10.1097/AOG.0000000000001910 28178064

