# Supplementary material for: Contributing factors of birth asphyxia in Thailand: a case–control study
Source: BMC Pregnancy Childbirth. 2023 Aug 15;23:584. doi: 10.1186/s12884-023-05885-y (PMC10426058; doi:10.1186/s12884-023-05885-y)
Supplement: Supplementary file 1 — Additional file 1. Intrapartum Care Record Form. [file 12884_2023_5885_MOESM1_ESM.pdf]

Supplementary I

Intrapartum Care Record Form

Hospital Code....................Level……..Mother Code ....Newborn Code...................

GA………………Date of Birth…………………G….P…P...A…L……………………………..

Indicator	Item	Score	Note
1. Partograph	1.1 Partograph use?	2. yes<br>0. no	
2. Partograph from a referring facility	2.1 Partograph recorded before refer?	Yes<br>No<br>N/A	
3. Cervical Dilatation	3.1 Cervix dilation recorded during admission<br>- Cx. dilate…………….at time………..<br>- Time of onset…………………………<br>- Time of admission……………………	2. yes<br>0. no	
	3.2 Cervical recoding time during ≥4 cms. At…………………………..	2. yes<br>0. no	
	3.3 Cervix dilation recorded every 4 hours<br>- latent………………………….<br>- active………………………..	2. yes<br>1. partially<br>0. no<br>N/A if duration of delivery less than 4 hrs.	
4. Descending	4.1 Descending recorded?	2. yes<br>1. partially<br>0. no	
5. Contraction	5.1 Uterine contraction recorded every 30 min<br>If not, duration of record…………..	2. yes<br>1. partially<br>0. no	
6. Membranes	6.1 Membrane rupture recorded?<br>Time of rupture………………….	2. yes<br>0. no<br>N/A	
	6.2 Amniotic fluid characteristic recoded?<br>Type…………………………….	2. yes<br>0. no<br>N/A	
7. Fetal Heart Rate	7.1 Fetal Heart Rate recorded every 30 min?<br>- latent………………<br>- active………………	2. yes<br>1. partially<br>0. no	
8. Maternal monitoring	8.1 Blood pressure recorded during admission?	2. yes<br>0. no	
	8.2 Blood pressure recorded every 4 hours?	2. yes<br>1. partially	

Indicator	Item	Score	Note
		0. no<br>N/A if duration of delivery less than 4 hrs.	
	8.3 Pulse recorded during admission?	2. yes<br>0. no	
	8.4 Pulse recorded every 4 hours?	2. yes<br>1. partially<br>0. no<br>N/A if duration of delivery less than 4 hrs.	
	8.5 Temperature recorded?	2. yes<br>0. no	
9. Crossing the lines	9.1 Partograph lines cross alert line?	yes<br>no	
	9.2 Partograph lines cross action line?	yes<br>no	
	9.3 If crossing the alert or action line, care activities recorded?<br>- movement encouraging<br>- bladder clear<br>- uterine contraction stimulation<br>- refer or alert team	2. yes<br>0. no<br>N/A if not crossing the lines	
10. Fetal non-reassuring	10.1 Fetal non-reassuring during active phase?<br>- time of identification…………….....<br>- how to detected……………………..	yes<br>no	
	10.2 non-reassuring level recorded?<br>- level……………………………	2. yes<br>0. no<br>N/A if not	
	10.3 Intrauterine resuscitation recorded?<br>- ……..Position change<br>- ……..Oxygen<br>- ……..stop oxytocin<br>- ……..IV fluid loading<br>- ……..other……………………………	2. yes<br>0. no<br>N/A if not	
	10.4 Severe non-reassuring time to delivery less than 30 min?<br>- Type of birth…………………………..<br>- Duration of delivery…………………	2. yes<br>0. no<br>N/A if not	
11. Second stage	11.1 Fetal Heart Rate recorded?	2. yes	

Indicator	Item	Score	Note
	- Duration of record…………………….	0. no	
	11.2 Fetal Heart Rate recorded every 5 min if non-reassuring?	2. yes<br>1. partially<br>0. no<br>N/A if reassuring	
	11.3 Prolong second stage?<br>- duration of second stage……………..	yes<br>no	
	11.4 Care activities during prolong second<br>-………..upright position<br>-……….. clear bladder<br>-………...team alert<br>-…………other…………………………..	2. yes<br>0. no<br>N/A if not	
12. Outcomes	12.1 Newborn health recorded?<br>-time of delivery……………………….<br>-1 min APGAR………………………..<br>-5 min APGAR………………………..<br>-10 min APGAR………………………	yes<br>no	
	12.2 Asphyxia newborn was resuscitated in 60 min?	2. yes<br>0. no<br>N/A if APGAR >7	
	12.3 Resuscitation activities<br>-……...skin stimulation<br>-………warm and dry<br>-………airway<br>-………oxygen<br>-………ET tube<br>-………IV fluid<br>-………medication<br>-………chest compression	2. yes<br>0. no<br>N/A if APGAR >7	
