# Supplementary material for: Contributing factors of birth asphyxia in Thailand: a case–control study
Source: BMC Pregnancy Childbirth. 2023 Aug 15;23:584. doi: 10.1186/s12884-023-05885-y (PMC10426058; doi:10.1186/s12884-023-05885-y)
Supplement: Supplementary file 2 — Additional file 2. Asphyxia Risk Factors Record Form. [file 12884_2023_5885_MOESM2_ESM.pdf]

Supplementary II

Asphyxia Risk Factors Record Form

Hospital Code ……………..............................……Level…………….............................

Mother Code ....Newborn Code............................ GA………………………

Categories	Recodes		Note
I: Antepartum risk factors of birth asphyxia			
1. Maternal Age (years)			
2. Was mother a booked case?	Y	N	
- 1st criteria visit (< 12 wks)	Y	N	- GA at visit
- 2nd criteria visit (18±2 wks)	Y	N	- GA at visit
- 3rd criteria visit (26±2 wks)	Y	N	- GA at visit
- 4th criteria visit (32±2 wks)	Y	N	- GA at visit
- 5th criteria visit (38±2 wks)	Y	N	- GA at visit
3. Did the mother suffer from any of these conditions during pregnancy			
- Maternal hypertension	Y	N	
- Gestational diabetes	Y	N	
- Anemia	Y	N	
- Ante partum hemorrhage	Y	N	
- Pre-eclampsia	Y	N	
- Diabetes mellitus	Y	N	
- Placenta Previa	Y	N	
4. Was the mother treated with any of the drug given below			
- Glucocorticoid	Y	N	
- Diuretics	Y	N	
- Antimetabolites	Y	N	
- Ethyl alcohol	Y	N	
- Adrenergic drugs	Y	N	
5. Is the mother	Primigravida	Multigravida	
6. Difference between previous baby and current baby in Multigravida (years)			
7. Socioeconomic status (bath / month)			
8. History of abortion	Y	N	If yes, number of abortion…
9. Pre-pregnancy BMI	Weight…………kg.<br>Hight…………..m.<br>BMI………….kg/m2		
II. Intrapartum risk factors of birth asphyxia			
10. Presentation of fetus	Cephalic	Breech	
11. Mode of delivery	Normal vaginal delivery	Cesarean section	
	Vacuum extraction	Forceps Extraction	
12. If cesarean, anesthesia received by	General anesthesia	Spinal anesthesia	

Categories	Recodes		Note
mother during c-section was			
13. Is there any history of prolonged labor?	Y	N	
14. Delivery conducted by whom	Doctor	Nurse	
III. Fetal risk factors of birth asphyxia			
15. Were any fetal conditions suspected/ diagnosed during pregnancy			
- Multiple birth	Y	N	
- Polyhydramnios	Y	N	
- Oligohydramnios	Y	N	
- Meconium-stained amniotic fluid	Y	N	
- Abnormal heart rate or rhythm	Y	N	
- Acidosis (fetal scalp capillary blood)	Y	N	
- Decreased rate of growth (uterine size)	Y	N	
- Premature delivery	Y	N	
- Amniotic fluid surfactant test negative or intermediate within 24 hours of delivery	Y	N	
- Ultrasound guided gross abnormality	Y	N	
- Abnormal estriol levels	Y	N	
16. Was the child resuscitated?	Y	N	
17. If yes, then how was the newborn resuscitated			
- Suction	Y	N	
- Facial oxygen	Y	N	
- Bag + mask IPPV	Y	N	
- ET intubation IPPV	Y	N	
- Medications	Y	N	
- Vascular resuscitation	Y	N	
20. Did the neonate suffer any of the conditions given?			
- Cord strangulation around neck	Y	N	
- Significant fetal distress	Y	N	
21. Gestational age of the baby at birth			
- Pre-term	Y	N	
- Term	Y	N	
- Post term	Y	N	
22. Baby weight (grams)			
