# Supplementary material for: Contributing factors of birth asphyxia in Thailand: a case–control study
Source: BMC Pregnancy Childbirth. 2023 Aug 15;23:584. doi: 10.1186/s12884-023-05885-y (PMC10426058; doi:10.1186/s12884-023-05885-y)
Supplement: Supplementary file 3 — Additional file 3. Intrapartum Health Care System Questionnaire. [file 12884_2023_5885_MOESM3_ESM.pdf]

Supplementary III

Intrapartum Health Care System Questionnaire

Hospital Code ……………………...................…Level……………….................................

Services structure	number
1. Number of pregnant women utilized intrapartum services in 1 year	
1.1 High risk pregnancies without delivery	
1.2 Vagina delivery	
1.3 Ceasarean section	
2. Bed	
3. Attended bed, Delivery bed and 2 hrs. postpartum observe bed	
4. Full time provider	
- Nurse-midwives	
- General physician	
- Obstetrician	
- Pediatrician	
- Anesthetrician	
5. Obstetric emergency team	
- Obstetrician	
- Experience nurse-midwives	
- Pediatrician	
- Anesthetrician	
6. Training	
- Partograph use	
- Cardiotocograph interpretation	
- Fetal non-reassuring	
- Obstetric emergency	
- Neonatal resuscitation	
7. Specialized nurse	
8. Advance Practice Nurse	
9. Nursing care model	
10. Nurse staff allocation	
- Day	
- Afternoon	
- Night	
10.1 Nurse experience	
- Novice	
- Intermediate	
- Expertise	
11. Nurse work hour	Shift per week (hour)
- Lowest	
- Highest	
- Average	
12. Number of fetal outcomes	

**Services structure**	**number**
- Vaginal birth	
- Mulpresentation	
- Vacuum extraction	
- Forceps extraction	
- Ceasarean	
- Live birth	
- Stillbirth	
- Asphyxia (APGAR at 1 min $\leq$ 7)	
- Severe asphyxia (APGAR at 5 min $\leq$ 3)	
